# Inhibition of protein kinase CK2 by CX-5011 counteracts imatinib-resistance preventing rpS6 phosphorylation in chronic myeloid leukaemia cells: new combined therapeutic strategies

**DOI:** 10.18632/oncotarget.7569

**Published:** 2016-02-22

**Authors:** Valentina Salizzato, Christian Borgo, Luca Cesaro, Lorenzo A. Pinna, Arianna Donella-Deana

**Affiliations:** ^1^ Department of Biomedical Sciences and CNR Institute of NeuroSciences, University of Padova, 35131 Padova, Italy

**Keywords:** chronic myeloid leukaemia, imatinib-resistance, protein kinase CK2, rpS6, CX-5011

## Abstract

Chronic myeloid leukaemia (CML) is a myeloproliferative disorder promoted by the constitutive tyrosine kinase activity of Bcr-Abl oncoprotein. Although treatment with the Bcr-Abl-inhibitor imatinib represents the first-line therapy against CML, almost 20-30% of patients develop chemotherapeutic resistance and require alternative therapy. Here we show that a strong hyper-phosphorylation/activation of ERK1/2, Akt Ser473, and 40S ribosomal protein S6 (rpS6) is detectable in imatinib-resistant KCL22 and K562 CML cells as compared to the -sensitive cell variants. In imatinib-resistant CML cells, high concentration of imatinib is required to strongly inhibit Bcr-Abl, ERK1/2 and Akt Ser473 phosphorylation, but under these conditions the phosphorylation of rpS6, a common downstream effector of MEK/ERK1/2 and PI3K/Akt/mTOR pathways is only slightly reduced. By contrast, down-regulation of the protein kinase CK2 by the inhibitor CX-5011 or by silencing the CK2 subunits does not affect the activation state of MEK/ERK1/2 or PI3K/Akt/mTOR signalling, but causes a drop in rpS6 phosphorylation in parallel with reduced protein synthesis. CK2-inhibition by CX-5011 induces cell death by apoptosis and acts synergistically with imatinib or the MEK-inhibitor U0126 in reducing the viability of imatinib-resistant CML cells. The ternary mixture containing CX-5011, imatinib and U0126 represents the most effective synergistic combination to counteract CML cell viability.

These results disclose a novel CK2-mediated mechanism of acquired imatinib-resistance resulting in hyper-phosphorylation of rpS6. We suggest that co-targeting CK2 and MEK protein kinases is a promising strategy to restore responsiveness of resistant CML cells to imatinib.

## INTRODUCTION

Chronic myeloid leukaemia (CML) is a malignant myeloproliferative disorder of primitive pluripotent stem cells arisen from the chromosomal translocation [t(9;22)(q11;q34)], which gives rise to the *BCR-ABL1* fusion oncogene [1]. The tyrosine kinase activity of the resulting Bcr-Abl oncoprotein is necessary and sufficient for initiation, maintenance and progression of CML phenotype. Bcr-Abl triggers the activation of multiple pathways that cooperate to drive critical pro-survival advantage counteracting cellular apoptosis [2, 3]. Targeting Bcr-Abl activity by the selective and potent Bcr-Abl-inhibitor imatinib mesylate (Gleevec) has become the front-line therapy for CML patients. However, up to one third of patients acquire resistance or intolerance to imatinib and require alternative therapies [4, 5]. Although resistance to imatinib is mainly caused by genetic and/or functional alterations of Bcr-Abl oncoprotein, Bcr-Abl-independent mechanisms of imatinib-resistance have been also described, including CML stem cell quiescence, expression of multi-drug-resistant phenotype or activation of alternative oncogenic pathways upstream or downstream of Bcr-Abl [4, 5, 6, 7]. The knowledge of these mechanisms has provided the opportunity for a second generation of dual-specific inhibitors or combination therapies to overcome the limitation of imatinib-resistance [5, 8, 9].

Protein kinase CK2 is a highly conserved and constitutively active Ser/Thr protein kinase, which is usually present as a tetramer composed of two catalytic (α and/or α’) and two regulatory (β) subunits. This protein kinase is distributed in all subcellular compartments, where it phosphorylates a huge number of protein substrates implicated in fundamental cellular processes [10–12]. CK2 is abnormally elevated in many human cancers, where it plays a global role as an anti-apoptotic and a pro-survival agent [13–15]. This protein kinase has never been described as the main driver of malignant transformation in cancer cells but rather as a critical cooperating partner of tumorigenic pathways able to potentiate the effect of known oncogenes [11]. CK2 up-regulation has been also shown in cancer cells displaying resistance mechanisms, either related to a multidrug resistance phenotype or induced by specific drugs [8, 16, 17]. We have recently demonstrated that, in imatinib-resistant CML LAMA84 cells, both expression and activity of CK2 are up-regulated as compared to imatinib-sensitive cells and that CK2 co-operates with Bcr-Abl to maintain the CML phenotype. Consistently, the combination of CK2-inhibition and imatinib-treatment acts synergistically in counteracting LAMA84 cell viability [8]. Interestingly, sensitization towards imatinib observed upon CK2-inhibition occurs also in imatinib-resistant CML cell lines that do not express abnormally high CK2 protein level [8].

This study provides new insights into molecular mechanisms of imatinib-resistance related to CK2 in CML KCL22 and K562 cell lines, where the drug treatment does not induce an up-regulation of the kinase. Particular attention is focused on MEK/ERK1/2 and PI3K/Akt/mTOR survival pathways to highlight a potential CK2-mediated hyper-activation induced by imatinib-resistance. The potent and very selective CK2-inhibitor CX-5011 is used in combination with imatinib and the inhibitor of MEK to define new therapeutic strategies able to overcome imatinib-resistance.

## RESULTS

### Hyper-phosphorylation of ERK1/2, Akt Ser473 and rpS6 is associated with imatinib-resistance in CML cells

**Figure 1:**
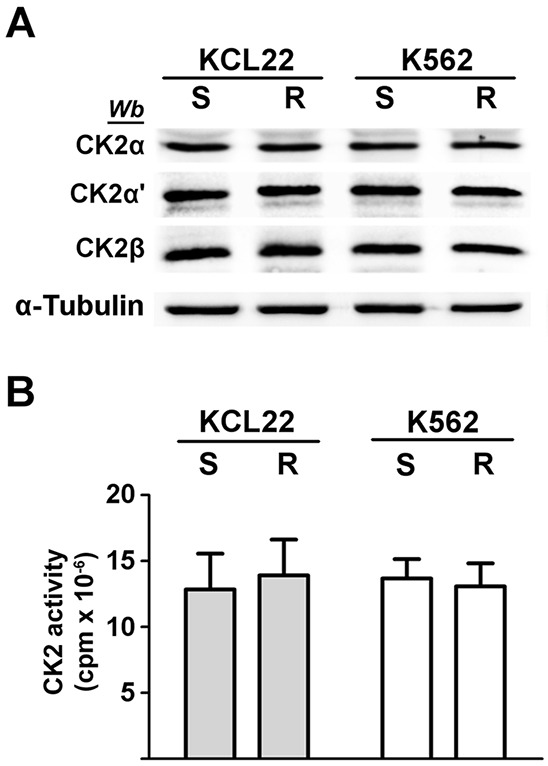
Analysis of CK2 expression and activity in CML cells KCL22 and K562 cells, either imatinib-sensitive (S) or -resistant (R) were lysed. **A.** Lysate proteins were analysed by western blot with the indicated antibodies. α-Tubulin was used as a loading control. Figure is representative of at least five separate experiments. **B.** Cellular kinase activity of CK2 was tested *in vitro* in a phosphorylation medium containing lysate proteins, [γ^33^P]ATP and the peptide-substrate RRRADDSDDDDD as detailed in Materials and Methods. Kinase activity is expressed as the amount (cpm) of ^33^P-phosphate transferred to the peptide per mg of lysate protein. Reported values are means ± SD of four separate experiments.

**Figure 2:**
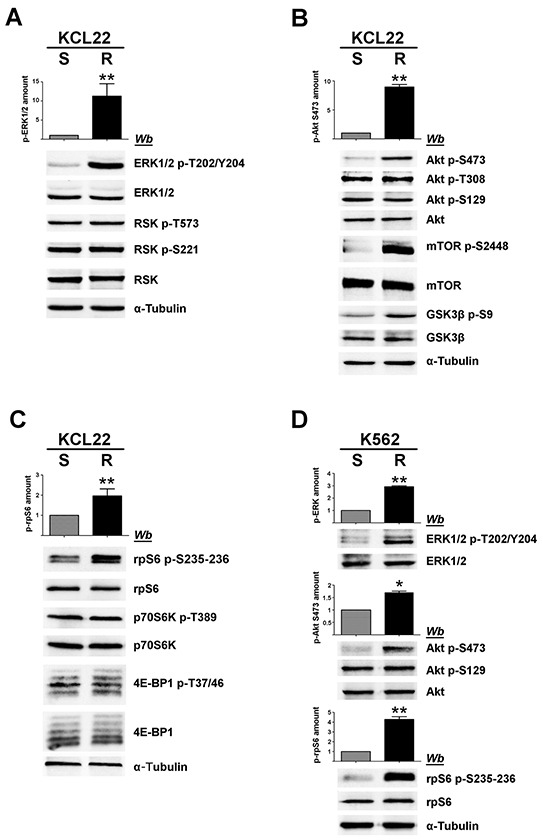
Analysis of MEK/ERK1/2 and PI3K/Akt/mTOR pathways in imatinib-sensitive and -resistant CML cells **A, B, C.** S- and R-KCL22, and **D.** S- and R-K562 cells were lysed and lysate proteins were analysed by western blot with the indicated antibodies. Means of the densitometric values ± SD relative to the phosphorylation extent of ERK1/2 p-T202/Y204 (A, D), Akt p-S473 (B, D) and rpS6 p-S235-236 (C, D) are reported above the immunostained bands. Densitometric values of imatinib-sensitive cells were arbitrarily set equal to 1.0. Figure is representative of at least four separate experiments. *p < 0.05 or **p < 0.01 *vs* imatinib-sensitive cells.

CML KCL22 and K562 cells, either sensitive (S) or resistant (R) to imatinib were used in our investigation. In these cell lines, resistance to imatinib is not caused either by *BCR*-*ABL1* gene amplification or by mutations of the Bcr-Abl kinase domain [18] or by expression of the efflux drug transporter P-glycoprotein [19]. Western blot analysis of cellular lysates demonstrates that imatinib-resistant KCL22 and K562 cells contain comparable protein-level of CK2 subunits (Figure 1A), [8] and similar CK2 activity in comparison with the sensitive variants (Figure 1B). To highlight a potential role played by CK2 in resistant CML cell lines, we focused our attention on the two Bcr-Abl downstream survival pathways MEK/ERK1/2 and PI3K/Akt/mTOR [2, 3], which are also under the control of CK2 at different levels [20–23] (see Figure 8). Expression and phosphorylation/activation state of different proteins involved in these two signalling cascades were first compared in imatinib-sensitive and -resistant KCL22 cells by means of western blot analysis with specific phospho-antibodies. Whereas the level of total ERK1/2 is similar in S- and R-KCL22 cells, imatinib-resistant cells are characterized by a striking hyper-activation of ERK1/2 as demonstrated by the hyper-phosphorylation of the residues Thr202/Tyr204 [24] (Figure 2A). ERK1/2-catalyzed phosphorylation of RSK Thr573 is the first step for its activation [25]. The phosphorylation level of RSK Thr573 as well as that of RSK Ser221, which triggers the kinase activation [25], are similar in the two cell variants (Figure 2A), suggesting that RSK pathway is not affected by the anomalous hyper-activation of ERK1/2 induced by imatinib-resistance.

PI3K/Akt/mTOR pathway was then analysed. It is well known that Akt becomes active following the phosphorylation of Thr308 and Ser473 [26]. Akt can be further regulated by the CK2-mediated phosphorylation of Ser129, which prevents the dephosphorylation of Akt Thr308 maintaining the kinase in its active conformation [21, 27]. Our analysis of Akt at Ser473 reveals that this activating residue is more phosphorylated in R-KCL22 that it is in S-KCL22 cells (Figure 2B). Consistently, an increased phosphorylation of the Akt targets mTOR Ser2448 and GSK3β Ser9 is observed, confirming the occurrence of an anomalous Akt activation induced by imatinib-resistance. In contrast with Akt Ser473, the activation state of Akt Thr308 is unaffected by the drug-resistance (Figure 2B). The constitutive phosphorylation of Akt Ser129, catalysed by CK2, is also similar in imatinib-sensitive and -resistant KCL22 cells (Figure 2B) consistent with the comparable CK2 protein expression and activity found in the two cell variants (Figure 1).

Next, we analysed the 40S ribosomal protein S6 (rpS6), a downstream effector of both MEK/ERK1/2 and PI3K/Akt/mTOR signalling cascades. In particular, we examined the phosphorylation extent of the residues Ser235 and Ser236 that can be phosphorylated by both RSK and p70S6K [28] (see Figure 8). Phosphorylation of rpS6 appears almost 2-fold higher in R-KCL22 cells as compared to the parental cell line, while its protein-level is similar (Figure 2C). The activation state of RSK has been shown to be similar in S- and R-KCL22 cells (Figure 2A). Therefore we analysed the phosphorylation of Thr389, which is required for p70S6K activation and is catalysed by mTORC1 (mTOR complex 1) [29], demonstrating that also the activation state of p70S6K is comparable in S- and R-KCL22 (Figure 2C). These results suggest that the hyper-phosphorylation of rpS6 is not directly mediated by an increased activation of its kinases RSK and p70S6K (see Figure 8). As in the case of p70S6K, the other main mTORC1-substrate 4E-BP1 (the eukaryotic translational initiation factor 4E binding protein 1) is similarly expressed and phosphorylated in the two cell variants (Figure 2C).

The hyper-activated proteins identified in R-KCL22 cells were then analysed in K562 cells comparing their phosphorylation extent in imatinib-sensitive and -resistant cell variants. Figure 2D shows that, also in these CML cells, imatinib-resistance is associated with an increased phosphorylation of ERK1/2 Thr202/Tyr204 (more than 2-fold), Akt Ser473 (almost 2-fold) and rpS6 (about 5-fold).

### rpS6 phosphorylation is independent of Bcr-Abl in imatinib-resistant KCL22 cells

**Figure 3:**
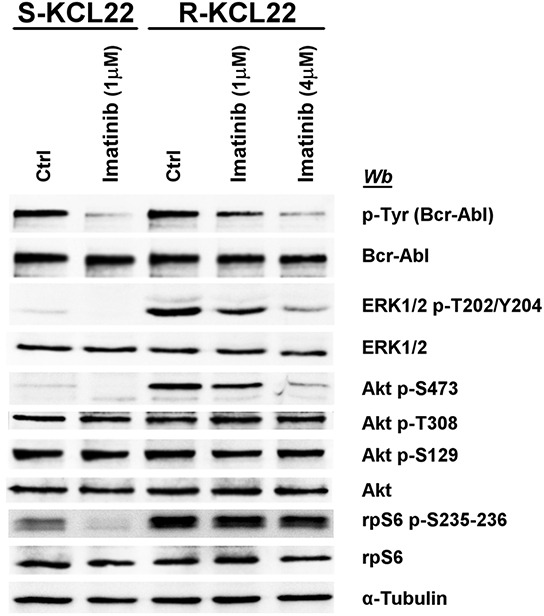
Effect of imatinib on MEK/ERK1/2 and PI3K/Akt/mTOR pathways in KCL22 cells S- and R-KCL22 cells, treated with DMSO (Ctrl) or with the indicated amounts of imatinib for 4 h, were lysed and lysate proteins were analysed by western blot with the indicated antibodies. Figure is representative of four separate experiments.

The inhibitory effect of imatinib on the pathways deregulated by the drug-resistance was compared in S- and R-KCL22 cells. In sensitive cells the treatment with 1 μM imatinib for 4 h is sufficient to efficiently counteract Bcr-Abl auto-phosphorylation and to block its downstream signalling as demonstrated by the abrogation of ERK1/2, Akt Ser473 and rpS6 phosphorylation (Figure 3). As expected, in R-KCL22 cells a higher concentration of imatinib is required to promote a strong inhibition of the phosphorylation extent of Bcr-Abl and its downstream effectors ERK1/2 and Akt Ser473, while Akt phospho-Thr308 is not inhibited even by this drug concentration. The additional observation that high imatinib concentration does not reduce Akt phospho-Ser129, demonstrates that the drug is not effective on CK2 activity (Figure 3). Of note, Bcr-Abl inhibition only marginally reduces (about 15%) the phosphorylation level of rpS6, outlining a Bcr-Abl-independent mechanism that sustains cell survival and cooperates to increase the DC_50_ (concentration inducing 50% of cell death) value for imatinib from 0.9 μM in S-KCL22 cells to about 40 μM in R-KCL22 cells (data not shown).

### rpS6 phosphorylation is dependent on CK2 in imatinib-resistant KCL22 cells

In an attempt to identify the protein kinase(s) responsible for rpS6 hyper-phosphorylation occurring in imatinib-resistance, R-KCL22 cells were treated with the inhibitors of the well-known pathways leading to this protein phosphorylation. We also used CX-5011 (Glixx Laboratories, South Borough, MA), a derivative of CX-4945, the first clinical stage inhibitor of CK2 for the treatment of solid tumours [30]. CX-5011 was preferred over CX-4945 for its comparable potency and higher selectivity [12].

**Figure 4:**
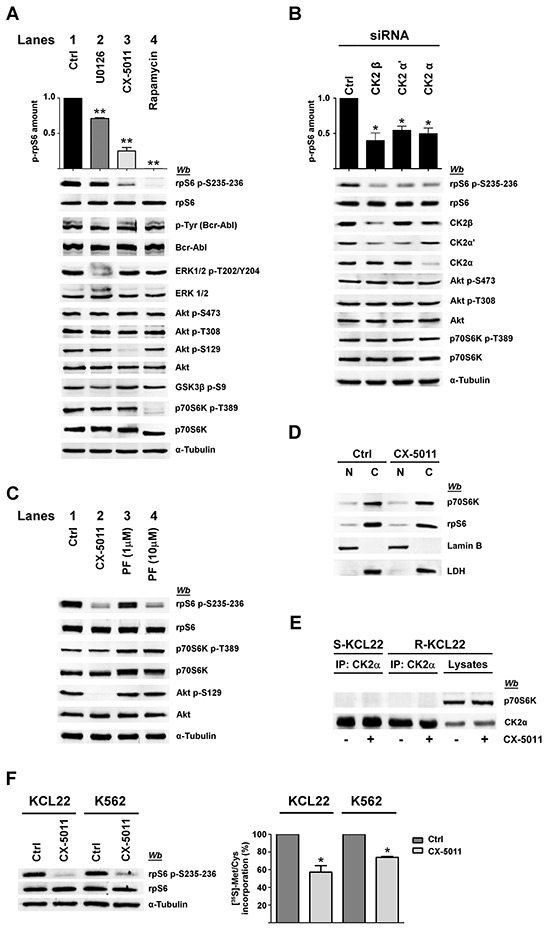
Effect of CK2 down-regulation by CX-5011 or siRNA on rpS6 phosphorylation and protein synthesis in imatinib-resistant CML cells **A.** Imatinib-resistant KCL22 cells, treated with DMSO (Ctrl), U0126 (10 μM), CX-5011 (3 μM) or rapamycin (20 nM) for 4 h, were lysed and lysate proteins were analysed by western blot. Means of the densitometric values ± SD relative to the phosphorylation extent of rpS6 p-S235-236 are reported above the immunostained bands and expressed in comparison with the control value, which was arbitrarily set equal to 1.0. Figure is representative of at least four separate experiments **B.** R-KCL22 cells were transfected with non-specific siRNA (Ctrl), or CK2β, CK2α’ or CK2α specific siRNAs for 72 h and lysed. Lysate proteins were analysed by western blot. Means of the densitometric values ± SD relative to the phosphorylation extent of rpS6 p-S235-236 are reported above the immunostained bands and expressed in comparison with the value obtained in cells transfected with non-specific siRNA, which was arbitrarily set equal to 1.0. Figure is representative of three separate experiments of transfection. **C.** R-KCL22 cells were treated with DMSO (Ctrl), 3 μM CX-5011, 1 or 10 μM PF-4708671 (PF). Cells were then lysed and lysate proteins were analysed by western blot with the indicated antibodies. **D.** R-KCL22 were pre-treated with either DMSO (Ctrl) or 3 μM CX-5011 for 4 h and then nuclear (N) and cytoplasmic (C) fractions were isolated as detailed in Materials and Methods. 20 μg of protein extracts were analysed by western blot with antibodies raised against p70S6K, rpS6, lamin B (nuclear marker), lactate dehydrogenase (LDH) (cytosolic marker). Figure is representative of three separate experiments. **E.** S-KCL22 and R-KCL22 cells were pre-treated with DMSO or 3 μM CX-5011 for 4 h and lysed. Lysate proteins (450 μg) were immunoprecipitated (IP) with anti-CK2α antibody and analysed by western blot. Aliquots of R-KCL22 lysate proteins (40 μg) were loaded on the right of the gel. Figure is representative of three separate experiments. **F.** R-KCL22 and R-K562 cells, pre-treated with DMSO (Ctrl) or 3 μM CX-5011 for 4 h, were treated with cycloheximide and pulsed in the presence of [^35^S]-L-Met/Cys protein labelling mix as described in Materials and Methods. Cells were lysed and 10 μg of radioactive proteins were subjected to SDS-PAGE and blotted. Membranes were analysed by western blot (left panel) and by Cyclone Plus Storage PhosphorSystem (right panel) to quantify the metabolic labelling, which is expressed as percentage relative to control values. Data represent mean values ± SD obtained from three independent experiments. *p < 0.05 or **p < 0.01 *vs* control values.

MEK-inhibitor U0126, which strongly reduces ERK1/2 phosphorylation/activation [31], only partially (about 20%) counteracts rpS6 phosphorylation in R-KCL22 cells (Figure 4A, compare lanes 1 and 2), while cell treatment with CX-5011 causes a drop in rpS6 phosphorylation of about 65% (Figure 4A, lane 3). The effect of CX-5011 on CK2 activity is confirmed by the almost abrogation of the CK2-mediated Akt Ser129 phosphorylation. Under our experimental conditions, however, CK2-inhibition does not counteract the activities of Bcr-Abl, ERK1/2 nor that of Akt as demonstrated by the comparable phosphorylation extent of Akt Ser473 and Thr308, and of Akt target GSK3β Ser9. Furthermore, CX-5011 treatment does not affect the activation state of p70S6K as indicated by its phosphorylation extent at Thr389 (Figure 4A, lane 3). As expected, cell treatment with rapamycin, an inhibitor of mTORC1 [32], almost abrogates both the phosphorylation of rpS6 and that of p70S6K Thr389 as also suggested by the kinase faster migration in SDS/PAGE [33] (Figure 4A, lane 4). Rapamycin treatment, however, does not inhibit the phosphorylation/activation of the other analysed protein kinases.

Collectively taken, these observations indicate that in imatinib-resistant KCL22 cells: i) rpS6 phosphorylation is only slightly mediated by MEK/ERK1/2 signalling; ii) the CK2-inhibitor CX-5011 strongly inhibits rpS6 phosphorylation under conditions that do not affect the activation state of either MEK/ERK1/2 or PI3K/Akt/mTOR/p70S6K pathway; iii) the mTORC1-inhibitor rapamycin abrogates the phosphorylation of p70S6K and of its substrate rpS6; iv) two independent pathways strongly prevent the phosphorylation of rpS6.

To further support the specific role played by CK2 in imatinib-resistance, we performed RNA-interference experiments knocking down the expression of the different CK2 subunits in R-KCL22 cells. Down-regulation of either the regulatory (β) or catalytic (α and α’) subunits is associated with a drop of rpS6 phosphorylation (Figure 4B). As observed with the pharmacological inhibition of CK2 (Figure 4A, lane 3), silencing CK2 activity does not affect the phosphorylation extent of Akt Ser473 and Thr308 nor that of p70S6K Thr389, confirming that PI3K/Akt/mTOR signalling is not influenced by the protein kinase down-regulation.

To investigate more deeply the potential interplay occurring between CK2 and p70S6K in mediating rpS6 phosphorylation, R-KCL22 cells were treated with inhibitors specific for the two protein kinases. Figure 4C confirms that CK2-inhibition by CX-5011 strongly counteracts rpS6 phosphorylation without affecting the activation state of p70S6K associated with Thr389 phosphorylation (compare lanes 1 and 2). PF-4708671 (PF) is a potent and selective inhibitor of p70S6K that induces an enhancement of the mTOR-catalysed phosphorylation of p70S6K at Thr389 [34] (Figure 4C, lanes 3,4). Cell treatment with 10 μM PF-4708671 strongly inhibits rpS6 phosphorylation, while it does not affect the CK2 activity as indicated by the phosphorylation extent of Akt Ser129, which is comparable to that of control (Figure 4C, lane 4). These results demonstrate that, in imatinib-resistant KCL22 cells, inhibition of either CK2 or p70S6K almost abolishes rpS6 phosphorylation, while the inhibition of one kinase does not influence the activity of the other.

It has been shown that CK2 phosphorylates p70S6K at Ser17 without affecting its kinase activity, but regulating its nuclear-cytoplasmic shuttling by enhancing its nuclear export in NIH3T3 cells [22]. To analyse the occurrence of a similar regulatory mechanism in CML cells, nuclear and cytoplasmic fractions were isolated from imatinib-resistant KCL22 cells treated with DMSO or CX-5011. 20 μg of protein fractions (corresponding to 20% of total protein extracted from nuclei and only 3.3% of cytoplasmic proteins) were then examined by western blot. Figure 4D shows that in control cells most of p70S6K is located in the cytoplasm of the leukemic cells, where also rpS6 is mainly detectable. Cell treatment with CX-5011 does not influence significantly the distribution of either p70S6K or rpS6, ruling out the hypothesis that CK2-inhibition might counteract rpS6 phosphorylation by affecting the translocation of its kinase p70S6K.

Since the report by Panasyuk et al. demonstrated by transfection experiments that p70S6K is also a binding partner of CK2 [22], we analysed the potential interaction occurring between the two proteins in imatinib-sensitive and -resistant KCL22 cells. Figure 4E shows that p70S6K does not co-immunoprecipitate with CK2 in the two cell variants either treated or not with CX-5011, excluding that, in imatinib-resistant cells, CK2 might influence rpS6 phosphorylation by interacting with p70S6K.

To further analyse the mechanism underlying the regulation of rpS6 phosphorylation by CK2, we examined whether the phosphorylation of the canonical rpS6 phospho-sites might be influenced by a potential CK2-catalyzed phosphorylation of the ribosomal protein at different site(s). To this purpose the protein was immunoprecipitated from R-KCL22 cell lysates, dephosphorylated by lambda phosphatase and subsequently phosphorylated *in vitro* in the presence of [γ^33^P]ATP by recombinant CK2 holoenzyme. The outcome that rpS6 is not phosphorylated by CK2 (data not shown) suggests that the kinase does not act directly on the ribosomal protein.

### CK2-inhibition affects protein synthesis efficiency in imatinib-resistant KCL22 and K562 cells

Since rpS6 phosphorylation is essential for protein synthesis and cell growth [35], we verified whether CK2-inhibition could affect the rate of the global protein synthesis by performing metabolic labelling experiments. Imatinib-resistant KCL22 and K562 cells, pre-treated with DMSO or CX-5011 for 4 h, were pulsed in the presence of a radiolabelled methionine and cysteine medium mix before quantification of total protein labelling. As expected, cell treatment with CX-5011 strongly decreases rpS6 phosphorylation, whereas the level of total rpS6 protein is unaffected (Figure 4F, left panel). Compared to controls, CK2-inhibition reduces the protein synthesis of about 43% and 25% in R-KCL22 and R-K562 cells, respectively (Figure 4F, right panel).

### Cell treatment with CX-5011 inhibits KCL22 cell viability by apoptosis induction

**Figure 5:**
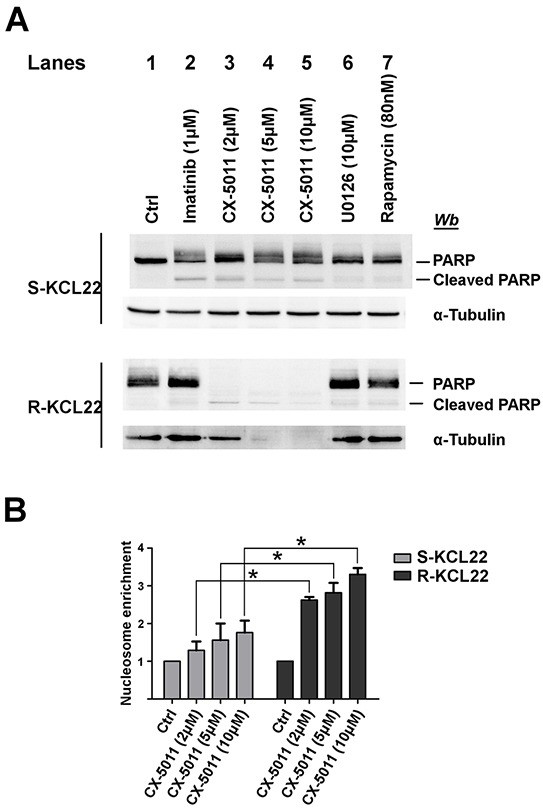
Analysis of apoptosis induction by CK2-inhibition in KCL22 cells **A.** S- and R-KCL22 cells, treated with DMSO (Ctrl) or with the indicated inhibitors for 48 h, were lysed and lysate proteins were analysed by western blot with the indicated antibodies. PARP antibody recognizes both full length and cleaved PARP. Figure is representative of four separated experiments. **B.** S- and R-KCL22 cells were treated with DMSO (Ctrl) or the indicated concentrations of CX-5011 for 4 h. Apoptosis was then tested using the Cell Death Detection Elisa kit as described in Materials and Methods. Nucleosome enrichment in treated cells was expressed in comparison with the control value, which was arbitrarily set equal to 1.0. Reported values are means ± SD of three independent experiments. *p < 0.05

It has been reported that the inhibitors of the MEK/ERK1/2 pathway, including U0126, can be classified as cytostatic but not as cytotoxic anticancer drugs [36] and that rapamycin does not inhibit the cell viability of imatinib-resistant cells by inducing apoptosis [37]. We analysed the apoptosis occurrence by comparing the cleavage of the caspase substrate PARP caused by treatment of KCL22 cells with imatinib, CX-5011, U0126 or rapamycin. 1 μM imatinib induces a substantial PARP cleavage in sensitive cells, while, as expected, it is ineffective on the imatinib-resistant variant (Figure 5A, compare lanes 1 and 2). On the contrary, treatment of imatinib-sensitive cells with CX-5011 up to 10 μM concentration induces only a partial PARP fragmentation, while 2 μM concentration is sufficient to cleave completely the protein in R-KCL22 cells and to induce a parallel proteolysis of cellular proteins, including α-tubulin (Figure 5A, lanes 3-5). This finding demonstrates that imatinib-resistant cells critically rely on CK2 for their survival. Addition of U0126 and rapamycin does not cause any appreciable effect on PARP cleavage in R-KCL22 cells (Figure 5A, lanes 6 and 7, respectively).

To further analyse the mechanism of cell death triggered by CX-5011, we evaluated the nucleosome formation in both imatinib-sensitive and -resistant KCL22 cells. Figure 5B shows that CK2-inhibition causes apoptosis in both cell lines with a cytosolic enrichment of nucleosomes much more pronounced in imatinib-resistant than in parental cell line, confirming that resistant cells become dependent on CK2 for their survival more than their sensitive counterpart.

### Co-targeting CK2 and MEK protein kinases synergistically overcomes the imatinib-resistance of R-KCL22 and R-K562 cells

**Figure 6:**
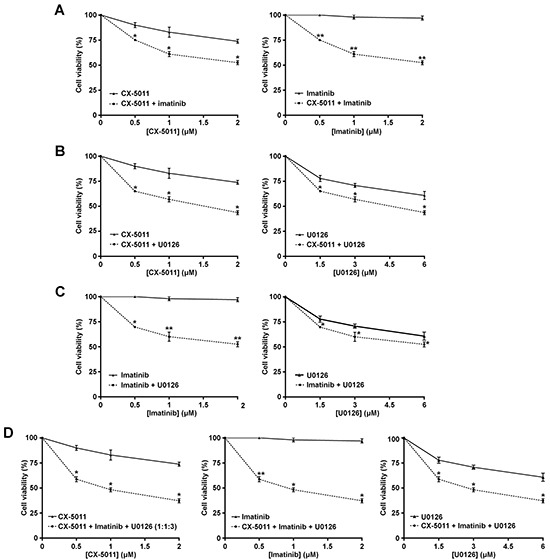
Effect of combined treatments with different inhibitors on R-KCL22 cell viability R-KCL22 cell viability was assessed by the MTT method after 48 h treatment with increasing concentrations of the following combinations of drugs, used at the indicated fixed ratio. **A.** CX-5011 and imatinib, 1:1; **B.** CX-5011 and U0126, 1:3; **C.** imatinib and U0126, 1:3; **D.** CX-5011, imatinib and U0126, 1:1:3. Cell viability was expressed as percentage of controls and plotted as function of each drug concentration. Mean ± SD values of at least five independent experiments are reported. *p < 0.05 or **p < 0.01 *vs* cells treated with a single inhibitor.

**Figure 7:**
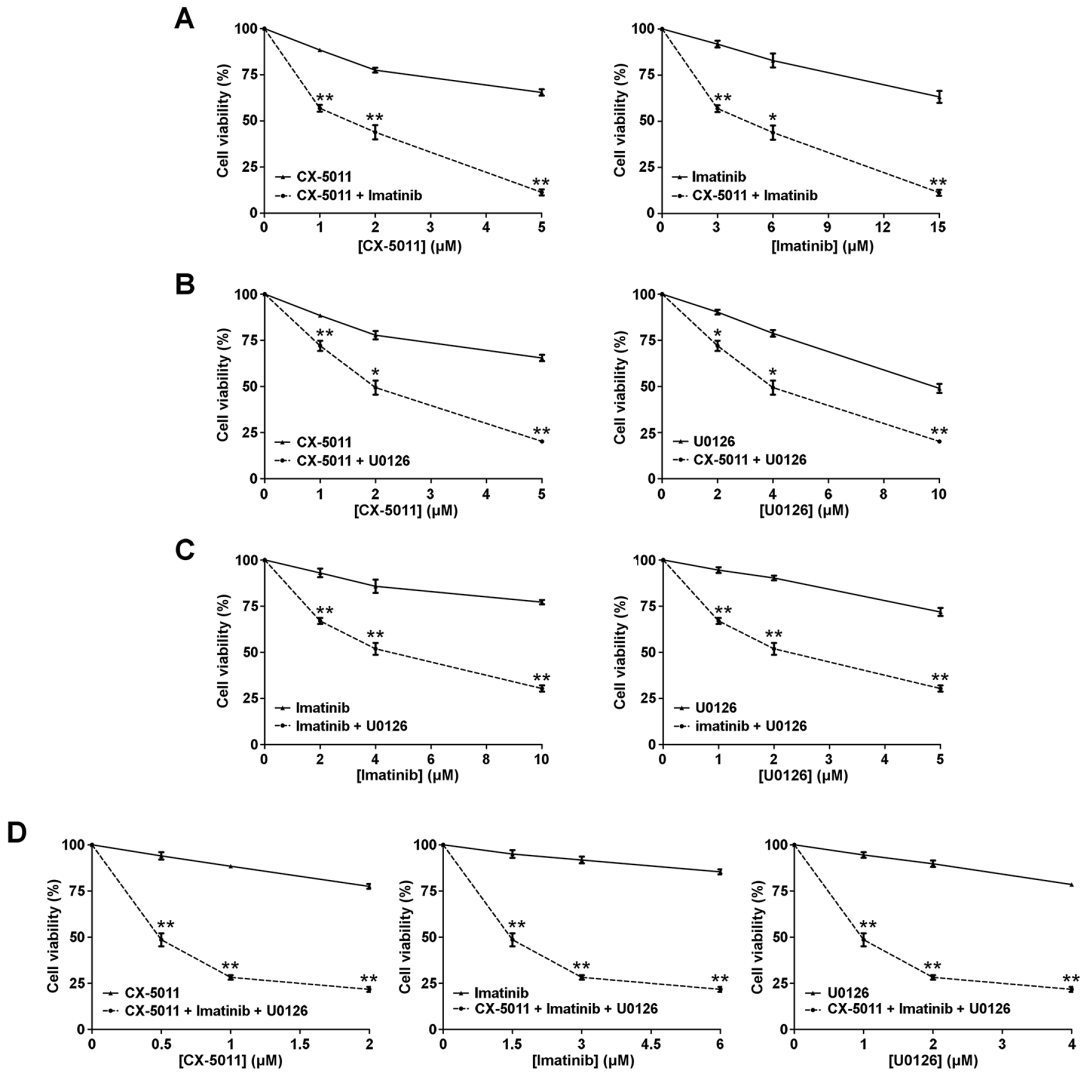
Effect of combined treatments with different inhibitors on R-K562 cell viability R-K562 cell viability was assessed by the MTT method after 48 h treatment with increasing concentrations of the following combinations of drugs, used at the indicated fixed ratio. **A.** CX-5011 and imatinib, 1:3; **B.** CX-5011 and U0126, 1:2; **C.** imatinib and U0126, 2:1; **D.** CX-5011, imatinib and U0126, 1:3:2. Cell viability was expressed as percentage of control and plotted as function of each drug concentration. Mean ± SD values of at least five independent experiments are reported. *p < 0.05 or **p < 0.01 *vs* cells treated with a single inhibitor.

We have recently found that co-treatment with imatinib plus CX-4945, a CK2-inhibitor of the same class of CX-5011 but less selective, causes a synergistic effect in reducing the cell viability of imatinib-resistant CML cell lines [8]. It has been also shown that the combination of imatinib and inhibitors of the MEK/ERK1/2 pathway may counteract the resistance to imatinib in CML cells [38, 39]. To unravel the potential effect of new drug combinations directed to different oncogenic targets activated by imatinib-resistance, CML cells were treated with imatinib, U0126 and CX-5011. We examined whether these drugs, added in different combinations to R-KCL22 and R-K562 cell cultures, could induce higher cytotoxicity as compared to the single treatments. Imatinib-resistant cells were treated for 48 h with inhibitors either alone, in binary or in ternary association, by increasing simultaneously the concentrations of the drugs added at fixed ratio [40]. Cell viability was determined by the MTT method and the viability was plotted as function of the single inhibitor concentrations (Figures 6 and 7). All the combined treatments promote a synergistic reduction of cell viability, as judged from the combination index (C.I.) calculated at the 50% of cell lethality, which denotes synergism if < 1 [40]. Our data show that CX-5011 strongly synergizes with imatinib with C.I. values of 0.50 and 0.32 in R-KCL22 and R-K562 cells, respectively (Figures 6A and 7A). When CX-5011 is used in association with U0126, a good synergistic effect is observed with C.I. values of 0.53 and 0.54 in R-KCL22 and R-K562, respectively, demonstrating that CK2-inhibition causes a substantial enhancement of U0126 effect (Figures 6B and 7B). The combination of imatinib with U0126 induces synergistic C.I. values of 0.60 and 0.33 in R-KCL22 and R-K562 cells, respectively (Figures 6C and 7C). Notably, the ternary mixture of CX-5011 with imatinib and U0126 represents the most effective synergistic combination to inhibit the viability of R-KCL22 (C.I. value = 0.35) and R-K562 cells (C.I. value = 0.17) and to restore responsiveness to low imatinib concentration (Figures 6D and 7D).

## DISCUSSION

**Figure 8:**
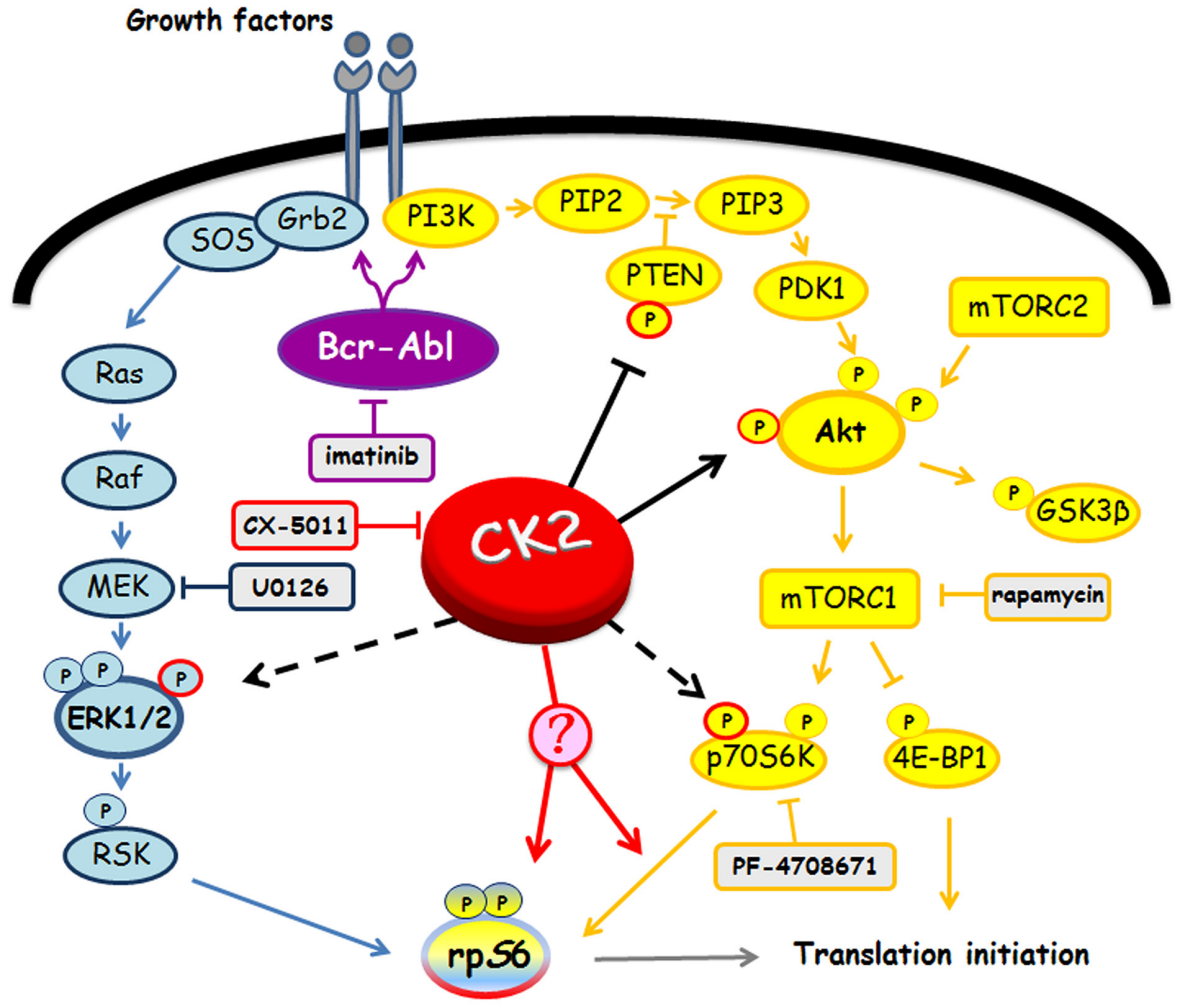
Involvement of Bcr-Abl and CK2 in MEK/ERK1/2 and PI3K/Akt/mTOR/p70S6K pathways Figure highlights signalling proteins affected by Bcr-Abl and CK2. Arrows and tee-arrows indicate substrate-protein activation or inactivation, respectively. Dashed arrows mean that CK2-phosphorylation affects the protein translocation. Red arrows indicate that, as demonstrated by our results, CK2 promotes rpS6 phosphorylation acting downstream of p70S6K.

We have previously demonstrated that the protein kinase CK2 is over-expressed in imatinib-resistant LAMA84 cells as compared to the -sensitive variant and that CK2 up-regulation strengthens the imatinib-resistance phenotype conferring survival advantage against imatinib [8]. In this study we analyse the CML KCL22 and K562 cell lines, characterized by a similar expression and activity of CK2 in imatinib-sensitive and -resistant cell variants (Figure 1), to gain information about unknown mechanisms of imatinib-resistance possibly related to CK2 even in the absence of its up-regulation. The involvement of CK2 in CML imatinib-resistance is investigated at multiple levels since this kinase does not act in a hierarchical way, but as a lateral player of the oncogenic signal transduction pathways [11]. Our results, on one hand, have led to the identification of a new Bcr-Abl-independent mechanism of resistance, and on the other, support the view that CK2 plays a general role in the oncogenic network related to CML imatinib-resistance, showing that its up-regulation is not a critical requirement for its contribution to imatinib-resistant phenotype. Analysis of KCL22 and K562 cells demonstrates that MEK/ERK1/2 and PI3K/Akt/mTOR pathways are up-regulated in imatinib-resistant cells as compared to the sensitive counterparts (Figure 2). Hyper-activation of MEK/ERK1/2 in imatinib-resistant CML cell lines has been previously described [38, 39, 41]. We also demonstrate an up-regulation of Akt revealed by the imatinib-induced hyper-phosphorylation of Ser473, one of the two canonical Akt activation sites, and confirmed by the hyper-phosphorylation of the Akt-substrates mTOR and GSK3β (Figure 2B and 2D). The finding that the Akt regulatory site Thr308 is similarly phosphorylated in imatinib-sensitive and -resistant KCL22 cells supports the notion that Thr308 and Ser473 fulfil essential, distinct, and non-overlapping functions as demonstrated by ablation or mutation of the two regulatory sites [42, 43]. Consistent with the activation of MEK/ERK1/2 and PI3K/Akt/mTOR signalling, the ribosomal protein S6, a common downstream effector of both pathways (see Figure 8), is hyper-phosphorylated at Ser235-236 in imatinib-resistant as compared to -sensitive KCL22 and K562 cells (Figure 2C and 2D). Cell treatment with U0126, however, demonstrates that rpS6 phosphorylation is only slightly (about 20%) under the control of MEK/ERK1/2 signalling in imatinib-resistant KCL22 (Figure 4A).

The specific role played by Bcr-Abl or CK2 on the pathways up-regulated upon imatinib-resistance was evaluated treating R-KCL22 cells with imatinib or CX-5011, respectively. As expected, higher concentration of imatinib is required in imatinib-resistant CML cells to counteract the constitutive activation of Bcr-Abl in parallel with the hyper-phosphorylation of ERK1/2 and Akt Ser473, while the same concentration is quite ineffective on the phosphorylation extent of rpS6 (Figure 3). On the contrary, CK2-inhibition strongly reduces rpS6 phosphorylation without affecting the phosphorylation of the residues responsible for the activation of Bcr-Abl, ERK1/2, Akt and p70S6K protein kinases (Figure 4A). Previous studies have demonstrated that inhibition of CK2 by CX-4945 decreases rpS6 phosphorylation in oncogenic cells as a consequence of the down-regulation of PI3K/Akt/mTOR signalling [44, 45]. Our results showing that CX-5011 and, under similar conditions, CX-4945 (data not shown) do not affect the activation state of MEK/ERK1/2 or PI3K/Akt/mTOR pathways suggest that an alternative regulatory mechanism mediated by CK2 acts on rpS6 phosphorylation in R-KCL22 cells. The outcome that CK2 plays a pivotal role in the regulation of rpS6 phosphorylation is also supported by RNA interference experiments. Knocking down CK2 subunit expression efficiently counteracts rpS6 phosphorylation without affecting the phosphorylation of Akt Ser473 and Thr308 and that of p70S6K Thr389 in imatinib-resistant KCL22 cells (Figure 4B).

Further studies are needed to get insight into the molecular process underlying the CK2 involvement in rpS6 phosphorylation in imatinib-resistant cells. CK2-inhibition strongly reduces rpS6 phosphorylation without affecting p70S6K, the main kinase active on this protein in R-KCL22 cells as indicated by cell treatment with rapamycin and PF-4708671. Therefore, we might hypothesize that the phosphorylation of rpS6 by p70S6K is under the control of a CK2-mediated regulatory mechanism. In this respect, our analyses rule out the hypotheses that CK2 might control rpS6 phosphorylation by interacting with p70S6K (Figure 4E) or affecting the nuclear *vs* cytoplasmic localization of p70S6K (Figure 4D). Moreover, our *in vitro* experiments demonstrate that the ribosomal protein is not a direct target of CK2 (data not shown). Another hypothesis is that imatinib-resistance might trigger an alternative p70S6K-independent and CK2-mediated pathway leading to rpS6 phosphorylation. On this point, evidence has been provided for an additional rpS6-phosphorylating kinase not identified so far [28].

Consistent with the role played by rpS6 in ribosome biogenesis and translation initiation [46], our results suggest that the hyper-phosphorylation of this protein in CML cells strengthens the resistant leukemic phenotype by promoting protein synthesis and cell growth. Indeed, down-regulation of rpS6 phosphorylation induced by CK2-inhibition is associated with a reduction of the protein synthesis efficacy as compared to the control in both KCL22 and K562 cells (Figure 4F). While the implication of CK2 in the regulation of protein synthesis is generally acknowledged [47], our results disclose a new Bcr-Abl-independent and CK2-mediated pathway associated with imatinib-resistance, which promotes protein synthesis thereby potentiating the CML cell survival.

The significant contribution of CK2 to the chronic myeloid leukaemia phenotype is confirmed by the apoptosis induction promoted by CX-5011 in KCL22 cells. We show that CX-5011 acts as a pro-apoptotic agent and that the inhibitor concentration required to induce apoptosis in imatinib-resistant KCL22 cells is much lower than that effective in sensitive cell line (Figure 5). This outcome is consistent with the concept that resistant cells are more dependent on CK2 activity for their survival than sensitive ones, although their CK2 level is not abnormally high. While our experiments were performed using CML cell lines, where imatinib-resistance is not associated with Bcr-Abl mutations [18], CK2-inhibition has been described recently to promote apoptosis induction in a human CML sample, where imatinib-resistance is due to Bcr-Abl T315I mutation [48].

The availability of different oncogenic targets hyper-activated by imatinib-resistance provided a criterion for assessing the efficacy of new drug combinations to counteract CML resistance. We found that all the binary associations between imatinib, U0126 and CX-5011, a derivative of a clinical stage inhibitor of CK2, act synergistically in reducing KCL22 and K562 cell viability. Of special interest is the combined inhibition of Bcr-Abl, CK2 and MEK protein kinases, which displays a potent and synergistic anti-proliferative effect. This ternary drug association is indeed particularly effective, restoring the sensitivity of imatinib-resistant cells to low imatinib concentration (Figures 6 and 7).

In conclusion, we provide evidence that imatinib-resistant KCL22 and K562 cells escape the drug effect by potentiating the cell proliferation mechanisms as well as the pro-survival pathways through up-regulation of ERK1/2, Akt Ser473 and rpS6 phosphorylation. We highlight a new regulatory pathway, mediated by CK2, involved in rpS6 phosphorylation in imatinib-resistant CML cells. We further show that the combined inhibition of Bcr-Abl, CK2 and MEK/ERK1/2 may afford a novel strategy to counteract CML imatinib-resistance by means of synergistic effects.

## MATERIALS AND METHODS

### Materials and antibodies

[γ^33^P]ATP was purchased from Perkin-Elmer (Waltham, MA). [^35^S]-L-methionine/cysteine protein labelling mix was from Hartmann Analytic (Braunschweig, Germany). Protease inhibitor cocktail was from Calbiochem (Darmstadt, Germany), while phosphatase inhibitor cocktails from Sigma-Aldrich (Dorset, U.K.). Imatinib mesylate was from Cayman Chemical (Ann-Arbor, MI), CX-5011 from Glixx Laboratories (South Borough, MA), rapamycin and U0126 from Selleck Chemicals (Houston, TX) and PF-4708671 from Sigma-Aldrich. RRRADDSDDDDD peptide was kindly provided by Dr. Oriano Marin (University of Padova, Italy). Anti-CK2α [8] antibody was raised in rabbit. Anti-c-Abl, anti-CK2α’, anti-mTOR, anti-Akt, anti-rpS6, anti-lamin B and anti-LDH antibodies were from Santa Cruz Biotechnology (Santa Cruz, CA), anti-phospho-tyrosine from Millipore Corporation (Billerica, MA) and anti-PARP from Roche (Basel, Switzerland). Anti-CK2β, phospho-RSK(S221), anti-RSK were purchased from Epitomics (Burlingame, CA). Antibodies against ERK1/2, phospho-ERK1/2(T202/Y204), phospho-RSK(T573), phospho-Akt(T308), phospho-Akt(S473), phospho-mTOR(S2448), GSK3β, phospho-GSK3β(S9), phospho-rpS6(S235-6), p70S6K, phospho-p70S6K(T389), 4E-BP1 and phospho-4E-BP1(T37/46) were from Cell Signalling Technology (Danvers, MA). Anti- phospho-Akt(S129) was purchased from Abcam (Cambridge, U.K.).

### Cell culture

KCL22 and K562 cell lines, either sensitive or resistant to imatinib, were maintained in RPMI 1640 supplemented with 10% fetal bovine serum, 2 mM L-glutamine, 100 U/ml penicillin and 100 mg/ml streptomycin in the absence (sensitive) or presence (resistant) of imatinib (3 μM or 0.6 μM for KCL22 and K562 cells, respectively).

### Cell lysis and western blot analysis

Cells were lysed by suspension (1 h at 4°C) in the lysis buffer A containing 20 mM Tris-HCl (pH 7.5), 1% Triton X-100, 10% glycerol, 1 mM EDTA, 150 mM NaCl and protease and phosphatase inhibitor cocktails. Protein concentration was determined by Bradford method. Proteins (20-30 μg) were subjected to 9% or 11% SDS-PAGE, blotted on Immobilon-P membranes (Sigma-Aldrich), processed in western blot with the indicated antibodies and developed using an enhanced chemiluminescent detection system (ECL). Immunostained bands were quantified by means of a Kodak-Image-Station 4000MM-PRO and analysis with Carestream Molecular Imaging software (New-Haven, CT).

### CK2 kinase activity assay

Different amounts of lysate proteins were separately incubated for 10 min at 30°C in 25 μl of a phosphorylation medium containing 50 mM Tris-HCl (pH 7.5), 100 mM NaCl, 12 mM MgCl_2_, 400 μM synthetic peptide-substrate RRRADDSDDDDD and 20 μM [γ^33^P]ATP (about 1000 cpm/pmol). Assays were stopped by absorption onto phosphocellulose filters. Filters were washed four times in 75 mM phosphoric acid [49] and analysed by a Scintillation Counter (PerkinElmer).

### RNA interference

Cells (1.5 × 10^6^) were transfected for 72 h with 50 nM CK2α siGENOME SMARTpool specific siRNA (Dharmacon, Lafayette, CO, USA) or CK2α’ [50] or CK2β (sequence target, GCCATGGTGAAGCTCTACT, was designed by Dr. M. Salvi) specific siRNAs. Aspecific siRNA siCONTROL riscfree#1 (Dharmacon) was used as a control. Cells were transfected using the transfecting reagent INTERFERin (Polyplus-transfection SA, Illkirch, France), according to the manufacturer's recommendations.

### Cell death detection by nucleosome enrichment quantification

Apoptosis was evaluated by means of the cell death detection Elisa kit (Roche, Mannheim, Germany), based on the quantification of nucleosomes present in the cytosol of the apoptotic cells, by measuring the absorbance at λ_405_–λ_490_, following the manufacturer's instructions. About 1 × 10^4^ cells were used for each determination.

### ^35^S -methionine/cysteine metabolic labelling

5 × 10^6^ cells were pre-treated for 4 h with vehicle DMSO (Ctrl) or 3 μM CX-5011 prior to add 100 μg/ml cycloheximide. After 2 h, the medium was replaced by methionine- and cysteine-free RPMI supplemented with 2 mM L-glutamine and 100 μg/ml cycloheximide. After 1 h, the medium was replaced by Met/Cys-free RPMI supplemented with 2 mM L-glutamine and 10 μCi/ml [^35^S]-L-methionine/cysteine protein labelling mix (Hartmann Analytic GmbH). After 1 h radiolabelled medium was removed and cells were washed and lysed as above described. Exposure to CX-5011 was maintained throughout the experiment. Similar amounts of radioactive lysates were subjected to SDS-PAGE and blotted. The membrane total labelling was measured using the Cyclone Plus Storage PhosphorSystem.

### Subcellular fractionation of R-KCL22 cells

R-KCL22 cells (7 × 10^6^), pre-treated with DMSO or 3μM CX-5011 for 4 h, were washed twice with PBS. Cells were then resuspended in 300 μl of ice-cold hypotonic buffer containing 20 mM Tris-HCl, pH 7.8, 1% (v/v) Nonidet P-40, and protease and phosphatase inhibitor cocktails. After 5 min, samples were diluted 1:1 with water and the solution passed through a 23 G needle 10 times using a 2 ml syringe. Samples were immediately centrifuged at 100 × *g* for 10 min at 4°C. Cytoplasmic fractions were collected as supernatants, while the pellets containing the nuclear fractions were extracted with 100 μl of lysis buffer A.

### Cell viability assay

Cell viability was detected by the method of MTT [3-(4,5-dimethylthiazol-2-yl)-3,5-diphenyltetrazolium bromide), incubating 4 × 10^4^ cells/100 μl in a 96-well plate under different conditions. 1 h before the incubation end, 10 μl of MTT solution (5 mg/ml in PBS) was added to each well. Incubations were stopped by addition of 20 μl of a pH 4.7 solution containing 20% (w:v) SDS, 50% (v:v) N,N-dimethylformamide, 2 % (v:v) acetic acid and 25 mM HCl. Plates were read at λ 540 nm absorbance, in a Titertek Multiskan Plus plate reader (Flow Laboratories, Sutton, U.K.). DC_50_ (concentration inducing 50% of cell death) values were calculated with Prism 4.0c software.

### Combined treatments

Effect of combined inhibitors was assessed by treating cells with increasing concentrations of drugs, added at fixed ratio, as indicated. The combination index (CI) [40] for the combined treatments were calculated with the software Calcusyn (Biosoft, Cambridge, U.K.); CI<1, CI=1 and CI>1 indicate synergistic, additive and antagonistic effects, respectively.

### Statistical analysis

Data are presented as means ± SD and mean differences were analysed using *t-test*. Values of *p < 0.05 or **p < 0.01 were defined as statistically significant.
